# Low-Cost Recruitment Approach: Utilizing Facebook Groups to Recruit COVID-19 Long-Haulers

**DOI:** 10.21203/rs.3.rs-4078924/v1

**Published:** 2024-03-29

**Authors:** Camryn Garrett, Shan Qiao, Cheuk Chi Tam, Xiaoming Li

**Affiliations:** University of South Carolina; University of South Carolina; University of South Carolina; University of South Carolina

## Abstract

**Introduction:**

The accessibility of social media (e.g., Facebook groups) presents long-haulers with the ability to connect with others with similar experiences and symptomology that are likely outside of their physical social networks. Social media sites may serve as promising platforms for research recruitment, public health campaigns, or interventions. The present study aims to assess, and comprehensively present, the effectiveness of a low-cost approach to recruitment through groups on Facebook within the context of a broader study of COVID-19 long-haulers.

**Methods:**

Facebook groups were searched using a variety of COVID-related terminology and included if they were in English, COVID-19 specific, public, and have or were approaching 1,000 or more members. Group administrators were either contacted for permission to post recruitment materials or posts were made and left pending administrator approval, depending on group settings. Group members were able to follow a link to the online survey platform (i.e., RedCap) where they provided informed consent and completed an online assessment of their COVID-19 experiences and psychosocial wellbeing. Upon survey completion participants were able to opt-in to a raffle-based incentive. The characteristics of the Facebook groups and demographic background of participants were assessed.

**Findings:**

Contacting administrators and posts made between January and March of 2022 within 17 COVID-19 specific groups yielded a sample size of 460 long-haulers. The groups relied upon for recruitment had a mean size of 21,022 (SD=45,645.3), most had three or more administrators (43%), and a majority were state specific (60%). The long-hauler participants enrolled from the posts had an average age of 32 years (SD=6.19), approximately split between men (48.91%) and women (50.22%), a majority white (70%), having earned a bachelor’s or postgraduate degree (63.48%), and reporting an annual income between $50,000 and $100,000 (56.09%).

**Discussion:**

The present study presents strengths and recommendations for survey recruitment through Facebook groups as a low-cost recruitment strategy that is easily targeted to populations with a specific health condition and allows users to complete online psycho-behavioral assessments off-site on a HIPPA compliant survey platform.

## Introduction

Despite the advancement of the COVID-19 pandemic into endemic stages, there remain a significant proportion of the population suffering from post-acute sequalae of SARS-CoV-2 Infection (PASC) [1]. Those who suffer from this form of chronic, long COVID are also known as COVID long-haulers. Common symptoms (i.e., loss of smell/ taste, post-exertional malaise, chronic cough, brain fog, thirst, palpitations, chest pain, fatigue, change in sexual desire or capacity, dizziness, gastrointestinal, abnormal movements, hair loss) experienced for a sustained period (i.e., 4 weeks or more) after COVID-19 infection are used to diagnose PASC [1]. Of the over 103 million cases of COVID-19 cases in the United States, 1 in 13 (7.5%) of those infected present symptoms of long COVID three months or more after infection [2].

Seeking connection and support in the online environment during COVID was motivated by stay-at-home orders and social distancing recommendations. The accessibility of social media presents long-haulers with the ability to connect with others with similar experiences and symptomology that are likely outside of their physical social networks. Systematic review studies have indicated the essential role of social media in help-seeking for mental health support, health information, and social networking during COVID-19 [3], [4]. As such, health literature has paid increasing attention to social media as a means for outreach, recruitment, and assessment.

Facebook is the largest social media platform worldwide with about 2.89 billion monthly users [5]. In the United States, about 70% of adults say they have ever used Facebook [6]. Of those who use Facebook, in the United States, 70% visit daily and 49% visit multiple times a day [6]. A key feature within the Facebook platform is the *Groups* function. Groups are defined by Facebook as a place for individuals to connect with others who share common interests for any topic or purpose [7]. Groups have three privacy levels: public, private, and secret [7]. Public Groups allow anyone, with or without joining, to see what is posted, commented, and shared within the group, as well as the list of members, moderators, and administrators. Private Groups allow only approved members of the group to see what is posted, commented, and shared within the group in addition to the list of members, whereas the list of moderators or administrators can be seen by anyone on Facebook [7]. Alternatively, secret groups are hidden so that the group is not visible anywhere on Facebook, including in the search bar, to anyone not invited to join or belonging to the group [7]. Users may join groups by clicking *join group* on any public group’s page, by requesting to join a private group, and through invitation to a secret group.

For the purposes of research, only public and private groups are of interest for recruitment due to their discoverability using the platform’s search function. Private groups often utilize screening questions, moderated by group administrators, to ensure those who are joining the group meet the criteria to participate. Users are individually limited to joining 6,000 groups [7]. If the limit is reached, then the user would need to leave joined groups before they would be able to join any new groups [7]. This extreme upper limit of group membership allows individual users to explore and join a variety of groups relevant to various interests, social groups, and phases of life. Due to their accessibility, Facebook groups present a unique platform through which members may connect over a shared interest, identity, or condition, which may be leveraged for the recruitment efforts of scientific research. The self-identification and self-selection of individuals into Facebook groups serve as a unique avenue through which public health surveillance and campaigns may be targeted and tailored, warranting further investigation into the effectiveness of Facebook group-based recruitment.

The utilization of Facebook groups for recruitment is a novel methodology but requires an investigation into the ability to obtain a representative sample, that is pertinent to the population of interest. One systematic review found that Facebook was the predominant social media website used for participant recruitment online, relying heavily on the paid, algorithmic-based advertisement feature offered through the platform [8]. Most studies of feasibility, sample representativeness and diversity, or cost effectiveness have been conducted using Facebook advertisements as the primary recruitment tool [9], [10], [11], [12]. The extant literature majorly quantifies engagement and cost-effectiveness through cost-per-click or cost-per-participant analyses, however, this metric has been largely applied, due to billing schemes, to advertisement-based, rather than group-based recruitment [9], [11], [13], [14].

Despite the high cost and limited insight into the influence of algorithms used in recruitment through paid advertisements, there has been limited work published considering the potential for recruitment through Facebook groups. Of the extant literature on Facebook group recruitment, a majority focuses on its functionality for hosting interventions (e.g., support groups), rather than as a tool for survey recruitment [15], [16], [17]. Emerging literature considering the potential for recruitment through a variety of methods on Facebook suggest promising results recruiting targeted populations through a study-specific page and engaging groups, but either consider only private groups or the utilization of paid post boosting within groups [13], [18]. Facebook groups present a unique opportunity to operationalize inclusion criteria and recruit a sample from a population self-identifying as members of the target population.

Despite the increase in studies utilizing online recruitment through Facebook, there persists no consensus on whether social media and group-based recruitment methods effectively recruit participants [11], [19]. Therefore, the present study, following recommendations within the literature, aims to assess, and comprehensively present, the effectiveness of a low-cost approach to recruitment, using Facebook groups, within the context of a broader study of COVID-19 long-haulers [20].

## Methods

### Group Searching

After the topic of interest was identified, a list of related keywords was compiled. These keywords included: Covid-19, Coronavirus, long-haulers, survivors, chronic, support, help, and recovery. Various combinations of these terms were utilized to search Facebook and identify potential groups for inclusion in the study. This search was conducted on the Facebook platform by navigating to the general groups page and searching the terms listed above. Here, as appropriate, additional keywords were utilized in searching due to their use in group names. An example of the inclusion of additional keywords would be the added searching of state-based groups as it was found users had created Covid-19 specific groups based on state residence.

### Group Inclusion Criteria Screening

Identified groups were then screened by their name and description. Groups were excluded if they were in a language other than English, private, or explicitly set in a country other than the United States. Inclusion criteria required groups to be in English, Covid-19 specific, public, and have or be approaching 1,000 or more members. Groups that were reasonably close to 1,000 were included to account for growth in membership between group identification, administrator contact, and data collection. Data collected about each group, based on availability, included the privacy setting, approximate number of members, number of administrators, location, group description, creation date, number of posts in the last month, group topics, and group rules. This information was utilized to identify groups eligible for recruitment (i.e., number of members, location, group description, group topics, and group rules), for recruitment contact (i.e., number of administrators), and to collect group activity (i.e., creation date, number of posts in the last month).

### Administrator Contact

Once all relevant groups were compiled into a dataset, the first listed administrator of each group was contacted using the platforms messaging functionality. The initial message followed a template to include a greeting with the name of the administrator recipient, followed by the researcher’s affiliated institution, researcher position, study topic, incentive, and ended with an inquiry as to if the administrator would approve a post about the study be made to the group page. An image of the research flyer was also sent following the message. If the administrator responded, their response (i.e., approve, deny, request for more information) was recorded. If there was no response within a week, a second administrator was contacted using the same template. If the administrator responded, their response was recorded. If there was no response within a week, a third, and final, administrator was contacted using the same template. This method of repeated contact to multiple administrators for requests to post was in efforts to increase the likelihood of a response. For groups with only one or two administrators, a short follow-up template was drafted and re-sent following the same week-long intervals. Following this timeline, requests for administrator approval would total three per group and take a minimum of three weeks.

### Advertisement Posts

**Figure 1:**
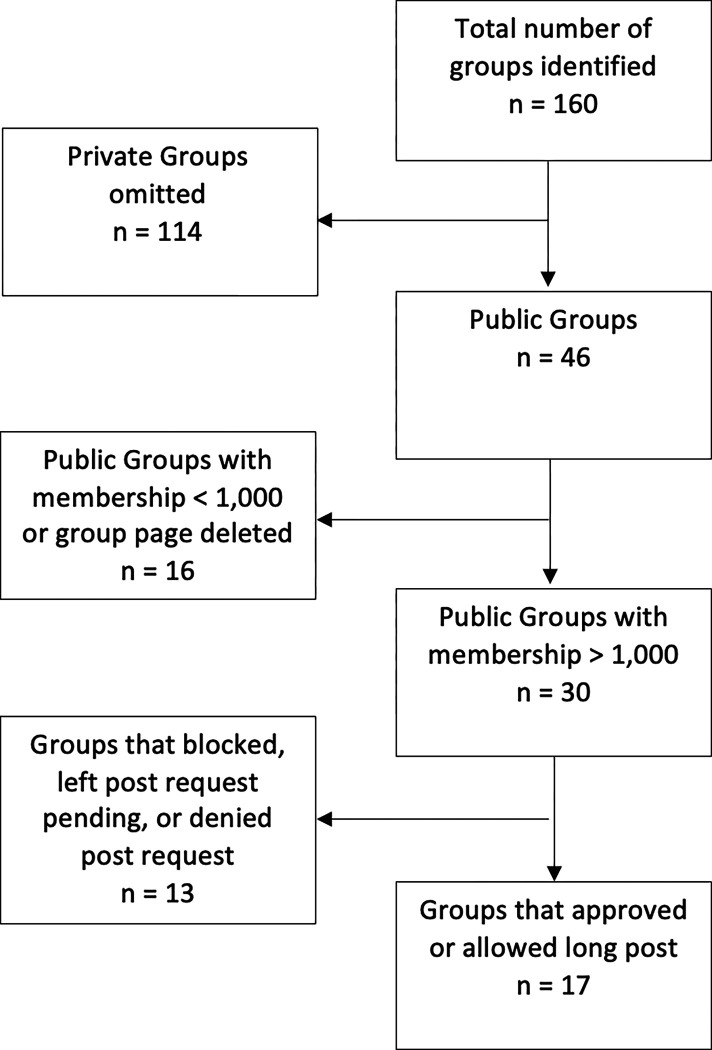
Groups Identified, Screened, Eligible, and Included for Recruitment

Upon approval, posts were made to participating Facebook groups. If there was no response from the attempts to contact administrators, then the post was made. The choice to post after no administrator response was based on the understanding that the post would undergo any privacy settings or screenings set in place by the group. Groups where there was no administrator response to messaging were found to, typically, have a moderation system in place where each post requires administrator approval. Posts were either published directly to the page or left pending until an administrator acted to approve or deny the post. Of the initial 160 COVID-centric Facebook groups identified, 30 met the inclusion criteria and 17 permitted a long-post be made to the group about the present study, of the 17 permitting the initial, long post only 10 permitted a second, shorter post (Fig. 1).

**Figure 2:**
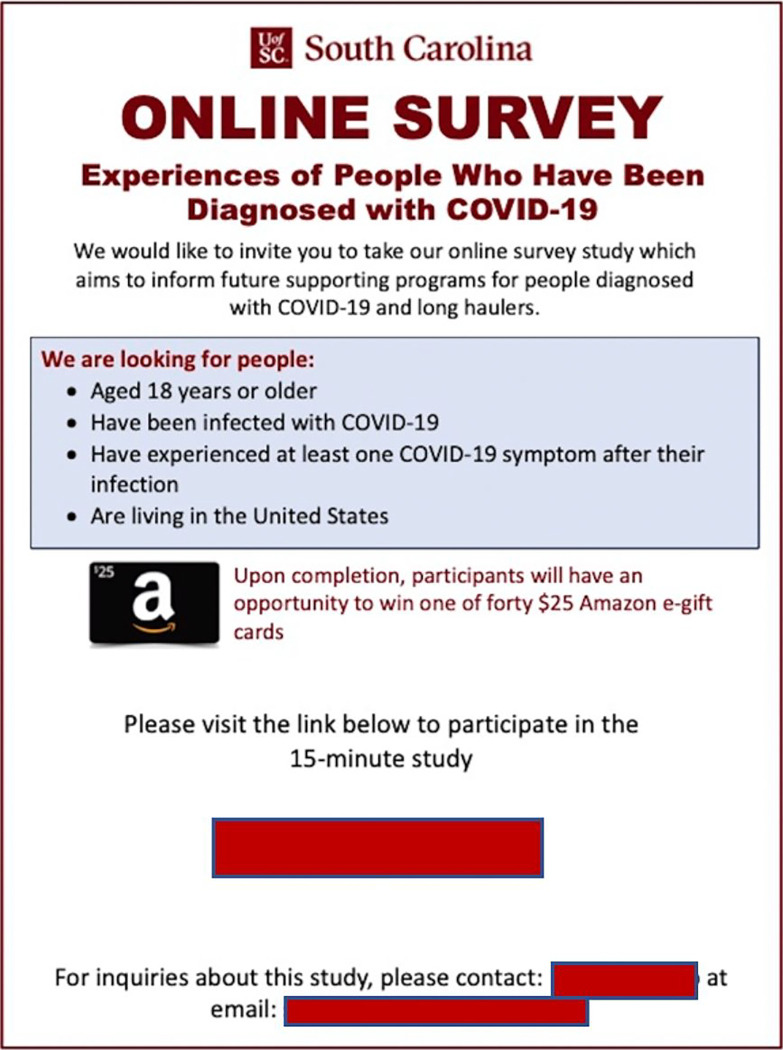
Study Flyer

The initial long post provided in-depth information about the study and required participants to click *see more* to access the survey link or follow the link provided on the attached flyer. The information detailed under *see more* included the inclusion criteria, institutional affiliation, aim of the work, incentive for participation, and survey hyperlink with an attached image of the study flyer (Fig. 2). The initial, long post made was:

“Have you had Covid-19 and experienced at least one Covid-19 symptom after infection? As a research team from the University of South Carolina, we are looking for individuals who are 18 or older, have been infected with Covid-19, have experienced at least one symptom after infection, and are living in the United States to complete our non-identifying survey. The aim of this work is to understand the experiences of people who have been diagnosed with Covid-19 and experienced persisting symptoms.

Upon completion of this 15-minute survey, all participants will be entered into a drawing to win one of forty $25 Amazon e-gift cards.

[Hyperlink to online survey platform]”

The second post was short and presented the survey hyperlink at first glance with only basic information of the inclusion criteria and an attached image of the study flyer. The second, short post made was:

“Have you had Covid-19 and experienced at least one Covid-19 symptom after infection? If so, complete our survey!

[Hyperlink to online survey platform]”

A standardized approach was used to ensure the same post was made to every group. An image was included in every post to draw user attention to the post while presenting the information in two formats: plain text and text within a colorful image. Each post had a lifespan of about one week where most responses were collected the day of and day after posting with responses dwindling up until the one-week point, after which no additional survey responses were recorded.

### Study Participants and Survey Administration

Individual survey respondents were recruited through their exposure to the study advertisements posted in Facebook groups of which they were a member. Upon clicking the survey hyperlink, participants were navigated from Facebook to the HIPPA compliant survey platform Redcap. Participants were provided additional detail regarding the study and potential risks. If after review they were still interested in participating, their navigation to the next page provided their informed consent. Utilizing Redcap’s survey flow logic, all participants were then screened according to the inclusion criteria for their eligibility. Those eligible to participate were those aged 18 years or older who self-reported ever having COVID, experienced symptoms due to infection, and were living in the United States. The survey measured constructs related to psychosocial wellbeing (e.g., depression, anxiety, post-traumatic stress, disorder, resilience, social support). Upon survey completion, participants were able to opt-in to a raffle-based incentive, from which 40 participants were randomly selected to receive a gift card incentive (i.e., USD 25 gift card).

### Findings

Following the methods described above, posts made to COVID-19 specific Facebook groups yielded a sample size of 460 long-haulers. This sample was obtained from posts made to each included group between January and March of 2022. The groups relied upon for recruitment had a mean size of 21,022 (SD = 45,645.3), most had three or more administrators (43%), and a majority were state specific (60%). Of the 17 groups that permitted the first, long post, only 10 permitted the second, short post. More posts were left pending or denied for the short post than the long post (Table 1).

The sample obtained had an average age of 32 years (SD = 6.19), approximately split between men (48.91%) and women (50.22%), a majority white (70%), having earned a bachelor’s or postgraduate degree (63.48%), and reporting an annual income between $50,000 and $100,000 (56.09%) (Table 2). If considering the cost of the raffle-based incentive relative to the number of participants, it is estimated that the average cost per participant was $2.17 although only 20 received monetary compensation for their time and efforts.

## Discussion

The sample collected in the present study generally aligns with the existing literature detailing the demographic profiles of COVID-19 long-haulers [21]. Emerging demographic profiles demonstrate that long-haulers, as identified by their symptoms rather than diagnosis, tend to be older (i.e., 53.7, SD = 21.0), female (59.7%), White (61.3%), Black (14.7%), and Hispanic or Latino (17.7%) [21]. Additional literature demonstrates mixed findings on the long-COVID burden experienced by certain racial or ethnic groups [22]. The Long COVID Household Pulse Survey from the CDC’s National Center for Health Statistics, based on estimates between October 18 and 30th 2023, found that of adults who ever had COVID, Hispanic or Latino (29.1%) adults, non-Hispanic White (24.9%) adults, and non-Hispanic Black (26.1%) adults represented those most affected [22]. Emerging literature, utilizing national level data (i.e., BRFSS, NHIS), has found similar trends of long-COVID burdens among older adults, females, Hispanic or Latino adults, and adults with less than a college degree [23]. In alignment with the emerging demographic profile of COVID-19 long haulers this study presents promising results to recruit samples of long-haulers among those most burdened (e.g., race, ethnicity, education) but require additional considerations to recruit a sample representative of long-haulers by age. Further approaches prioritizing a fully representative sample may rely on a quota-based sampling strategy to control for the over- or underrepresentation within certain stratifications.

The low-cost recruitment efforts detailed in the present study resulted in a total sample of 460 participants from advertisements posted in 17 COVID-centric, public Facebook groups. These group-based, online recruitment methods possess strengths and limitations that must be considered relative to the resources available, target population, as well as privacy and confidentiality responsibilities. Typical online recruitment relies on the advertising features of platforms but can become costly when paying per day, by reach, or by click. Traditional recruitment via paid platform advertisements may, themselves, be associated with concerns of reach and representativeness in addition to limitations of high cost without assurance of data completeness or quality. Broad advantages and disadvantages of social media based recruitment methods include their access to broad audiences, targeted recruitment, rapid recruitment, user engagement, and reduced costs in contrast to challenges to representativeness, privacy concerns and control, as well as limited access [24]. These advantages and challenges must therefore be weighed, as related to recruitment method, in study design.

Recruitment utilizing Facebook’s group features differentiates itself from the costly methods of recruitment through paid advertisements. Findings reporting the efficacy of paid advertising recruitment approaches praise abilities to reach specific demographic groups to align with target populations but result in a variable average cost per participant ranging from USD $1.88 to USD $4.21 [25]. A systematic review of Facebook recruitment for health research reports a median cost per participant of $14.41 [11]. Although the algorithmic distribution of advertisements may be useful to researchers aiming to recruit a particular demographic, the need for caution due to its nonrandom targeting has been called for in the literature [26]. Samples recruited through paid Facebook advertisements have been found to be partially representative, overrepresenting young white women, with issues of selection bias, based on language used in the advertisement, impacting engagement [11], [27]. The present findings demonstrate strength not only in the ability to target recruitment and tailor campaigns, beyond basic demographics, based upon group topics and membership identity (i.e., disease condition), but also in that its cost (i.e., $2.17 per participant) is similar to that of the lower end of costs associated with Facebook advertisement-based recruitment. Further, as the raffle-based incentive was determined by the study team with consideration of available resources and appropriate compensation, there is the potential for future studies to operationalize a similar approach while following an alternate incentive structure.

Generally, recruitment through Facebook groups, rather than advertisements, leverages certain principles of community engaged research where group administrators act as community leaders, gatekeepers, and stakeholders. Social media platforms present a unique setting through which to sample hard-to-reach populations. Use of social media methods for recruitment has been advantageously described as providing a sampling frame for populations where probability sampling has otherwise been inconceivable or infeasible [25]. In reimagining the role of Facebook groups for recruitment, various outcomes have been reported. One disease-specific RCT conducted during the COVID-19 pandemic found group recruitment to be effective as 478 individuals expressed interest in participating, of which 404 were eligible to participate and contacted, to which 135 responded and the first 100 to respond were utilized as the sample [28]. Mixed findings exist where although one study posted in 123 groups and obtained a sample size of 87 participants, another posted in 42 groups and obtained a sample size of 4,000 [29], [30]. In considering differences in the proportion of groups posted in and the resulting sample size, a possible explanation considers that recruitment may be contingent upon the size of the target population aimed to be sampled, relevant demographics, and intensity or required commitment of participants to complete study activities [29].

The findings of the present study demonstrate the recruitment of a diverse and sufficient sample and provide support for Facebook group recruitment methods that have been emergingly utilized within the literature. In considering Facebook recruitment methods, the researcher should consider, in relation to their research question, the target population (e.g., size, accessibility, presence on Facebook, number of public groups), available resources (e.g., recruitment costs, participant incentive costs), timeline (e.g., administrator contact schedule, posting schedule, time between posts), potential for bias (e.g., measuring social support amongst members in online groups), study design (e.g., RCT challenges vs. cross-sectional challenges), and data collection or intervention methods (e.g., confidentiality and privacy safeguards between participants, use of additional data collection methods to reduce the potential for anticipated bias) [24]. An additional consideration for researchers aiming to employ similar methods is the impact of the posting profile on user trust, where a newly created study profile may be perceived differently than posts made from a researcher’s personal profile, but raises questions related to privacy and professionalism necessary to ensure an appropriate distance between researchers and participants is maintained while also building trust [29].

The present study presents strengths of survey recruitment through Facebook groups as a low-cost recruitment strategy that is easily targeted to a specific population and allows users to complete the study survey activities off-site on a HIPPA compliant survey platform. Facebook group-based recruitment has demonstrated promise when recruiting for both qualitative and quantitative work. A potential limitation of this recruitment method is related to the impact on sampling and should be weighed against population access, sampling frames, and sampling strategies. An additional limitation of this method is the ever-changing nature of platform functions and user demographics where the functionality of groups is likely to change over time as those who are active on the site or utilize certain functions may also change over time. Recommendations for future use of this method are to consider including groups with membership less than 1,000 and including private groups after administrator approval. Anecdotally, it is recommended that posts be made when users are more likely to be active (e.g., outside of typical working hours, on weekends), to increase visibility, and to consider that the life of a post has been here found to be a week but may differ based on the level of activity and engagement in each group. If the group administrators are receptive to posts for health-related research recruitment they could be asked to pin posts to the top of the group page, also increasing visibility.

In summary, the present methods detailed to recruit study participants from Facebook groups provide supportive evidence for this low-cost recruitment method to target disease- or condition-specific groups that may be otherwise hard-to-reach. Future research should further investigate differences in representativeness and bias present in studies recruiting through Facebook groups versus advertisements as well as the influence of differences in perception of recruitment materials on the sample recruited (e.g., length of post, language used, perception of post maker). There also exists the potential to interview Facebook group creators and administrators regarding their perceptions of group purpose, group regulation, as well as member and post approval decision-making, especially as related to health-related research posts. There is a clear and present need for a review and meta-analysis of recruitment efficacy across and between various disease specific groups to inform future recruitment and intervention tailoring efforts to better understand the context and concerns of a variety of populations (e.g., stigma, belonging, diagnosis, chronic vs. temporary).

## Figures and Tables

**Table 1:** Group and Post Characteristics

Measure	Mean (SD) or n (%)

Group Size	21,022 (45,645.3)
*Median (QR)*	3,000 (1,650–13, 750)

Long Post Status	17 (56.7%)
Posted	6 (20%)
Pending	7 (23.3%)
Denied	

Short Post Status	10 (58.8%)
Posted	5 (29.4%)
Pending	2 (11.8%)
Denied	

Number of Participants Enrolled by Post Length	376 (81.7%)
Long	84 (18.3%)
Short	

Number of Group Administrators/ Moderators	10 (33.3%)
1	7 (23.3%)
2	13 (43%)
3+	

Geographic Region	12 (40%)
National	18 (60%)
State-Specific	

**Table 2:** Sample Characteristics

Variables	Mean (SD) or n (%)

Age, *mean (SD)*	32 (6.19)
18–24	24 (5.22%)
25–34	327 (71.09%)
35–44	88 (19.13%)
45–54	16 (3.48%)
55+	5 (1.09%)

Gender	231 (50.22%)
Women	225 (48.91%)
Men	4 (0.87%)
Other/ Prefer not to say	

Race/ Ethnicity (Check all that apply)	322 (70%)
White	79 (17.2%)
Black/ African American	41 (8.9%)
Hispanic/ Latino	4 (0.9%)
Asian	18 (3.9%)
Native American/ Alaskan Native/ Native Hawaiian/ Pacific Islander	

Education	14 (3.04%)
Highschool degree or equivalent	94 (20.43%)
Some college	60 (13.04%)
Associates degree	274 (59.57%)
Bachelor’s degree	18 (3.91%)
Postgraduate degree or above	

Annual Income	27 (5.87%)
< $10,000 to $24,999	164 (35.65%)
$25,000 to $49,999	258 (56.09%)
$50,000 to $100,000	11 (2.39%)
> $100,000	

## Data Availability

The data used to support the findings of this study are not publicly available but may be made available upon reasonable request.
